# IgA2^+^ B cells and IgA2 anti-dsDNA antibodies are selectively targeted by belimumab after rituximab therapy in systemic lupus erythematosus

**DOI:** 10.1016/j.xcrm.2025.102247

**Published:** 2025-07-23

**Authors:** Daniel McCluskey, Muhammad R.A. Shipa, Kashfia Chowdhury, Judith A. James, Laura A. Cooney, Michael R. Ehrenstein

**Affiliations:** 1 Department of Ageing, Rheumatology and Regenerative Medicine, University College London, London, UK; 2 Comprehensive Clinical Trials Unit, University College London, London, UK; 3 Oklahoma Medical Research Foundation, Oklahoma City, OK, USA; 4 Immune Tolerance Network, University of Michigan, Ann Arbor, MI, USA

**Keywords:** SLE, belimumab, rituximab, IgA2 anti-dsDNA antibodies, CD11c^+^Tbet^+^ age-related B cells, theragnostic biomarker, BAFF

## Abstract

No theragnostic biomarkers exist for systemic lupus erythematosus (SLE) to enable a precision medicine approach. Baseline serum IgA2 anti-double-stranded DNA (dsDNA) antibody levels are associated with response to combination belimumab after rituximab therapy in SLE (BEAT-lupus trial, ISRCTN 47873003). Analysis of the CALIBRATE trial (NCT02260934) confirms that baseline IgA2 anti-dsDNA antibody levels are specifically associated with response to belimumab after rituximab (odds ratio [OR] = 16.9, confidence interval [CI]: 2.8–101, compared to rituximab alone—CALIBRATE and BEAT-lupus combined data). IgA2 anti-dsDNA antibody levels decrease alongside IgA2 expression in plasmablasts only after this combination treatment. Increased serum B cell-activating factor (BAFF) levels are associated with rising IgA2 anti-dsDNA antibody levels after rituximab. IgA2 plasmablasts have increased BAFF receptor and interleukin (IL)-10 expression compared to IgA1 plasmablasts and have a distinct integrin profile implicating a gut mucosal origin. These findings validate IgA2 anti-dsDNA antibodies as a theragnostic biomarker of response and provide mechanistic insight into the selective targeting of IgA2^+^ B cells by combination belimumab after rituximab in SLE.

## Highlights

• IgA2 anti-DNA antibody levels predict response to belimumab after rituximab in SLE
• Belimumab after rituximab targets IgA2 plasmablasts and CD11c^+^Tbet^+^ B cells in SLE
• IgA2 and CD11c^+^Tbet^+^ B cells have increased expression of BAFFR and IL-10 in SLE

## Graphical abstract

**Figure:**
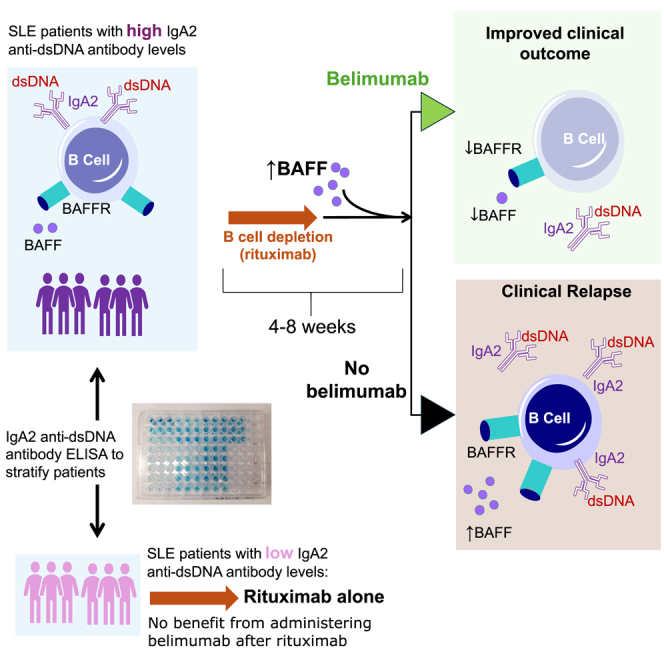

## Teaser

McCluskey et al. show that baseline serum IgA2 anti-dsDNA antibody levels are a companion biomarker of response to belimumab after rituximab in systemic lupus erythematosus. Increased BAFF receptor expression in IgA2-producing B cells augments their responsiveness to elevated BAFF levels post-rituximab treatment, rendering them susceptible to belimumab.

## Introduction

Systemic lupus erythematosus (SLE) is a complex autoimmune disease that can affect multiple organ systems and is characterized by the production of autoantibodies against double-stranded DNA (dsDNA), making therapies targeting B cells, such as the anti-CD20 therapy rituximab, an appealing therapeutic strategy.^1^^,^^2^ However, response to rituximab varies considerably, with no demonstrable benefit observed in placebo-controlled clinical trials.^2^^,^^3^ One plausible explanation is the increase in levels of B cell-activating factor (BAFF) after B cell depletion, which could cause disease flares.^4^ BAFF promotes B cell maturation and survival and is implicated in SLE pathogenesis.^5^

We previously published the BEAT-lupus trial, a placebo-controlled clinical trial comparing treatment with rituximab with or without the BAFF inhibitor belimumab in patients with SLE refractory to conventional therapy.^6^ BEAT-lupus showed that the combination of belimumab after rituximab significantly reduced IgG anti-dsDNA antibodies and the risk of severe flares compared to rituximab alone, but there was wide variation in response. Follow-up analyses of these results identified IgA2 anti-dsDNA antibodies as a potential predictive biomarker of response to belimumab after rituximab but not rituximab alone.^7^

There is an urgent need for biomarkers that predict response to targeted therapies in SLE because of disease heterogeneity, wide variation in response to treatment, and the high failure rate of targeted therapies in clinical trials. Even those treatments that demonstrated efficacy in clinical trials often yielded only modest overall benefit. Selecting the right treatment for the right patient through precision medicine has been described by one commentator as a “quest for the holy grail” for patients with SLE.^8^ Theragnostic biomarkers identified through analysis of a clinical trial require validation to progress their utility and can reveal the immune pathogenesis of a lupus endotype specifically responsive to a targeted therapy. No treatment-specific biomarker for SLE has overcome this barrier to development.

IgA2 is one of two subclasses of IgA that is less abundant than IgA1 in the serum (9:1 ratio; IgA1:IgA2) but is the dominant subclass in the gut.^9^ IgA2 is known to promote pro-inflammatory effects in neutrophils and macrophages, whereas IgA1 is less inflammatory.^10^ Our understanding of IgA2-producing B cells is still limited, possibly due to mice possessing only one IgA subclass. It remains unclear how IgA1 and IgA2 B cells are differentially influenced by rituximab and belimumab combination therapy in SLE. Here, we utilized the power of high-dimensional spectral flow cytometry to comprehensively profile B cells using samples from a second clinical trial, CALIBRATE,^11^ to validate the preferential effects of rituximab and belimumab on IgA2 identified in BEAT-lupus and expand our limited understanding of IgA2-producing B cells in SLE.

## Results

### The combination of rituximab and belimumab preferentially reduced plasmablasts in SLE

**Figure 1:**
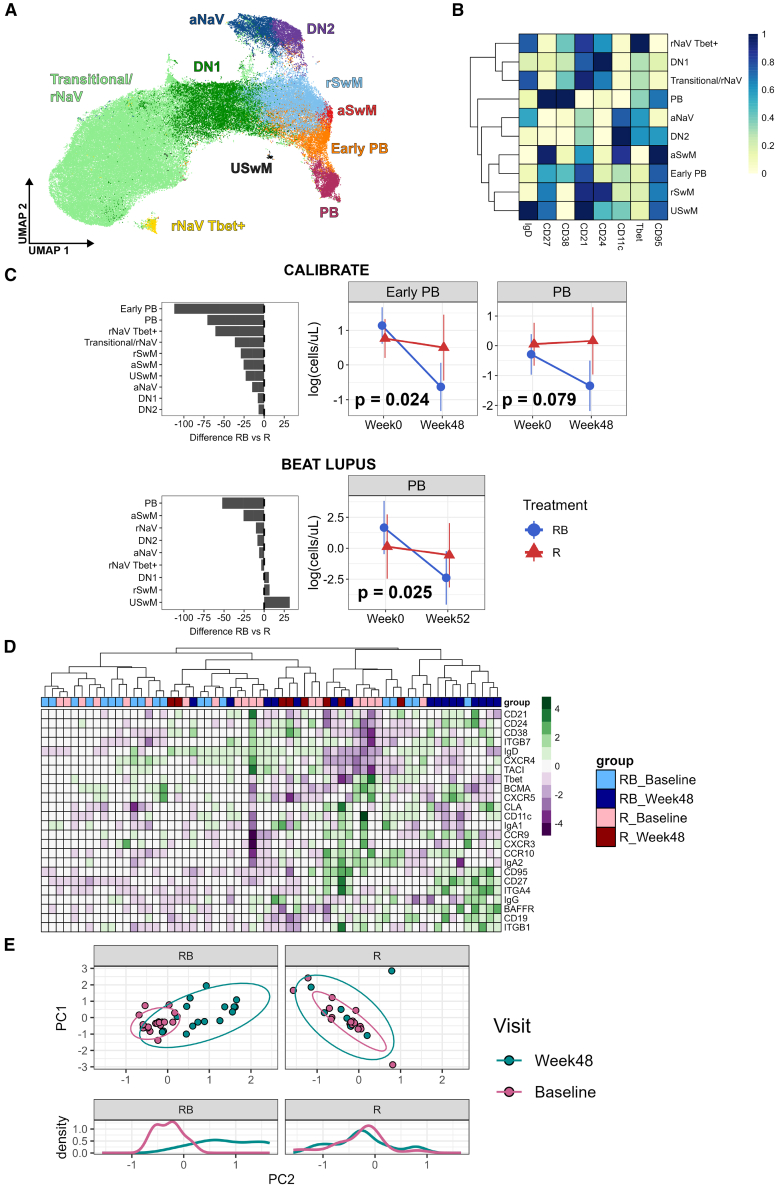
Clustering analysis of B cells (A) UMAP of clustered CD3^−^CD19^+^ B cells with canonical subsets labeled (CALIBRATE trial, *n* = 38). aNaV, activated naive; aSwM, activated switched memory; rSwM, resting switched memory; DN, double-negative; PBs, plasmablasts; rNaV, resting naive; USwM, unswitched memory. (B) Heatmap of key marker expression across each labeled cluster. (C) Left: difference in the percentage change of absolute number in each subset from baseline to week 48 (CALIBRATE, *n* = 38) and week 52 (BEAT-lupus, *n* = 35) between the two arms of the trials. Right: longitudinal measurement of log cells/μL in the plasmablast clusters. Mean plus 95% confidence intervals are shown. Linear mixed-effects model used to analyze difference between the two arms of the trial from baseline to week 48 (CALIBRATE) or week 52 (BEAT-lupus). (D) Heatmap of scaled median expression of each marker included in the flow cytometry panel from each sample (CALIBRATE). Top row is color-coded by treatment and visit. (E) Principal component analysis of patients at baseline and week 48 in each treatment arm (CALIBRATE). Bottom panel displays density histogram of PC2. RB, belimumab after rituximab; R, rituximab. Only *p* values < 0.1 are shown. See also Figure S1.

To investigate changes in B cell subsets after combination rituximab and belimumab therapy, peripheral blood mononuclear cells (PBMCs) from 38 patients (Table S1) recruited to the CALIBRATE trial were analyzed using 27-color spectral flow cytometry (Figure S1A). There was a reduction in the number of CD19^+^ B cells in participants treated with rituximab and belimumab compared to rituximab alone at week 24 (*p* = 0.025) and a trend toward a reduction at week 48 (*p* = 0.084) (Figure S1B). Clustering of 2,376,600 B cells resulted in the identification of 10 B cell subsets (Figure 1A). Canonical B cell subsets were annotated based on the expression of key markers (Figure 1B). The percentage change in the absolute numbers of each B cell subset was calculated at week 48 relative to baseline to determine the effects of belimumab following rituximab compared to rituximab alone. The difference between the two treatment arms with respect to the percentage change in absolute numbers at week 48 from baseline is displayed (Figure 1C, upper left panel). The plasmablast (PB) and early PB clusters showed the greatest difference between the treatment arms in the CALIBRATE trial (Figure 1C). The numbers of early PB were significantly reduced after combination therapy at week 48 compared to rituximab alone (*p* = 0.024). To validate these findings, results from a more limited 10-marker flow cytometry panel of PBMCs from the BEAT-lupus trial were available (*n* = 35), which tested belimumab after rituximab compared to rituximab alone in patients with SLE refractory to conventional therapy,^6^ and a conventional gating approach was applied. Consistent with the results from the CALIBRATE trial, the reduction in PB numbers in the belimumab after rituximab group at week 52 compared to baseline was the most prominent of all the changes in B cell subsets between the two arms of the BEAT-lupus trial (*p* = 0.025) (Figure 1C, lower panels).

We next examined whether the reduction in PB numbers was more pronounced in patients who responded to the combination therapy. Early PB numbers were significantly reduced (*p* = 0.002, *p* = 0.081 for PBs) in responders to the combination therapy in the CALIBRATE trial but not in patients who did not respond (Figure S1C). Changes in the rituximab-alone arm could not be calculated due to the lack of absolute count data for non-responding patients. A similar pattern was seen in the BEAT-lupus trial, with a trend toward a reduction in PBs (*p* = 0.079) in patients responding to combination therapy, whereas in the rituximab-alone arm, there was little difference between patients stratified by response (Figure S1C).

To interrogate the effects of the combination therapy on B cell phenotype more broadly, we assessed expression of all the markers in the panel across all samples from the CALIBRATE trial. Unsupervised hierarchical clustering revealed that samples form patients treated with the combination therapy, at the 48-week time point, tended to cluster away from the baseline samples, while samples from rituximab-alone patients clustered together across the baseline and 48-week time points (Figure 1D). Principal component analysis confirmed this observation, as patients treated with rituximab followed by belimumab showed greater cluster divergence between week 48 and baseline in contrast to those patients who only received rituximab (Figure 1E).

### The combination of belimumab and rituximab suppressed IgA2 but not IgA1 anti-dsDNA antibodies

**Figure 2:**
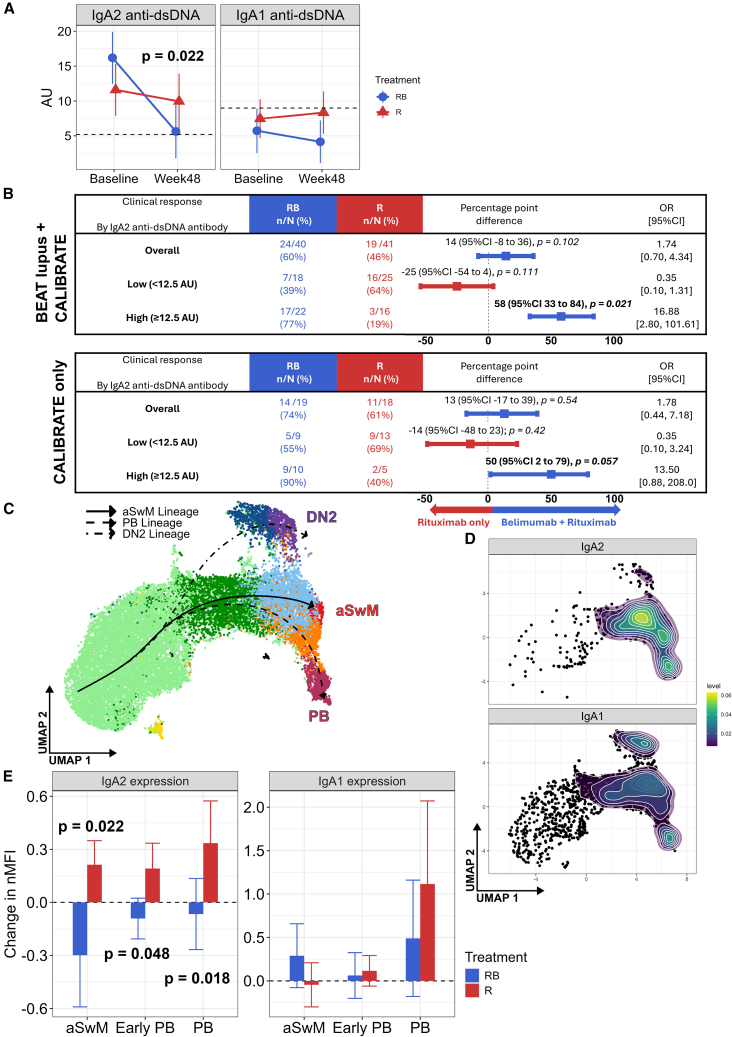
Serum IgA2 and IgA1 anti-dsDNA antibody levels and IgA2 and IgA1 expression in terminally differentiated B cell clusters (A) Serum IgA2 and IgA1 anti-dsDNA antibody levels at baseline and week 48 stratified by treatment (CALIBRATE trial). Mean plus 95% confidence intervals are shown. Dotted line represents upper limit of normal (mean +3SD), *n* = 37. Values are the mean of two technical replicates. *p* value comparing the difference at week 48 between the two arms of the trial adjusting for baseline values. Linear mixed-effects model used for analysis. (B) Baseline serum IgA2 anti-dsDNA antibody levels were categorized into high (≥12.5 AU) or low (<12.5 AU) levels and used as an effect modifier to predict clinical response at 48 (CALIBRATE) and 52 (BEAT-lupus) weeks in the two arms of the trials. The top panel combines patients from both BEAT-lupus and CALIBRATE, while the bottom panel only shows patients from the CALIBRATE trial. OR, odds ratio. (C) UMAP with three pseudotime trajectories overlaid (CALIBRATE). The rNaV/transitional cluster is the origin point for the trajectories. (D) UMAP of IgA2^+^ and IgA1^+^ B cells with kernel density overlaid. (E) Change in nMFI (expression) of IgA2 and IgA1 at week 48 compared to baseline in the terminally differentiated B cell clusters categorized according to treatment (CALIBRATE, *n* = 38). Mean plus 95% confidence intervals are shown. Linear mixed model used for all comparisons. RB, belimumab after rituximab; R, rituximab; nMFI, normalized mean fluorescence intensity. Only *p* values <0.1 are shown. See also Figures S2 and S3.

We previously reported that baseline serum IgA2 anti-dsDNA antibody levels were associated with response and are reduced following combination belimumab after rituximab therapy in the BEAT-lupus trial accompanied by a reduction in the frequency of IgA2 PBs.^7^ To validate these results, we measured serum IgA1 and IgA2 anti-dsDNA antibodies by ELISA using samples from the CALIBRATE trial. Serum IgA2 anti-dsDNA antibody levels were significantly reduced at week 48 after the combination therapy compared to those patients treated with rituximab alone (*p* = 0.022) (Figure 2A). There was no significant change in serum IgA1 anti-dsDNA antibody levels during the CALIBRATE trial (Figure 2A). Of note, serum IgA2 anti-dsDNA antibodies were increased compared to healthy controls, whereas serum IgA1 anti-dsDNA antibodies were close to normal values. There was no significant difference in total serum IgA2 and IgA1 antibody levels from baseline to week 48 between the two arms of the trial (Figure S2A).

We next compared the clinical outcome at 48 weeks with respect to achieving a complete or partial renal response in both arms of the CALIBRATE trial categorizing participants according to whether they had high or low serum levels of IgA2 anti-dsDNA antibodies at baseline as measured by ELISA using the cut-point value (10.7 AU) derived from the BEAT-lupus trial.^7^ 11 of the 12 patients with high IgA2 anti-dsDNA antibody levels at baseline achieved a partial or complete renal response when treated with combination rituximab and belimumab compared to 4 out of the 7 patients with high IgA2 anti-dsDNA antibody levels treated with rituximab alone (Δ35%, 95% confidence interval [CI]: −4 to 67); in contrast, there was a difference of 13% between the two arms of the trial with respect to achieving a major clinical response, analyzing the whole group irrespective of the biomarker (Figure S2B). By combining data from the CALIBRATE and BEAT-lupus trials, a better-performing cut-point of 12.5 AU was identified associated with a more significant interaction *p* value between high or low baseline IgA2 anti-dsDNA antibody levels measured in AU and response to treatment in each arm of the trial (*p* < 0.0001 compared to *p* < 0.001 using the 10.7 AU cut-point). Thus, the 38 (47%, of the 81) participants with a serum IgA2 anti-dsDNA antibody level equal or above 12.5 AU in the BEAT-lupus and CALIBRATE trials were 16.9 times more likely (CI: 2.8–101) to respond to belimumab after rituximab compared to rituximab alone (Figure 2B). The corresponding odds ratio (OR) of response in unselected patients treated with the combination compared to rituximab alone was 1.7 (CI: 0.70–4.34). For completeness, we then reanalyzed the CALIBRATE data alone using the 12.5 AU cutoff and found that the corresponding OR for patients with high baseline IgA2 anti-dsDNA antibody levels with respect to clinical response was 13.5 (CI: 0.88–208, *p* = 0.057) (Figure 2B). The response rate overall in the CALIBRATE trial (74% in the combination arm, 61% in the rituximab-only arm) was higher than in BEAT-lupus (48% in the combination arm, 35% in the rituximab-only arm^7^). The fewer non-responders in the CALIBRATE arm prompted us to calculate the cumulative prednisolone dose for each trial. Participants in the CALIBRATE trial received substantially more prednisolone compared to those recruited to BEAT-lupus (*p* < 0.001) (Figure S2C).

To understand the effect of combination therapy on IgA2-expressing B cells, we determined IgA2 expression (mean fluorescence intensity [MFI]) and percent positivity across all B cell clusters identified in Figure 1A at baseline (CALIBRATE trial). A representative scatterplot of IgA1 and IgA2 expression within PBs showed clear separation of IgA1 and IgA2 B cells (Figure S3A). The PB, early PB, and activated switched memory (aSwM) clusters had the highest IgA2 expression and contained the greatest proportion of IgA2 B cells (Figure S3B); therefore, we focused on these subsets for further analysis. Pseudotime analysis revealed three distinct differentiation trajectories, terminating at the double-negative 2 (DN2), aSwM, and PB clusters (Figure 2C). The highly differentiated nature of these subsets was confirmed by their high pseudotime scores (Figure S3C). IgA2 expression predominated in the switched memory and PB clusters, with little seen along the DN2 trajectory, while IgA1 expression was broader (Figure 2D).

We used IgA2 expression (MFI) as a surrogate for IgA2 production and investigated its change from baseline to 48 weeks in the CALIBRATE trial across the aSwM, PB, and early PB clusters. Strikingly, IgA2, but not IgA1, expression was significantly reduced at 48 weeks in all three clusters by the combination of rituximab and belimumab compared to rituximab alone (Figures 2E and S3D).

### BAFF receptor expression was higher in IgA2 compared to IgA1 PBs

**Figure 3:**
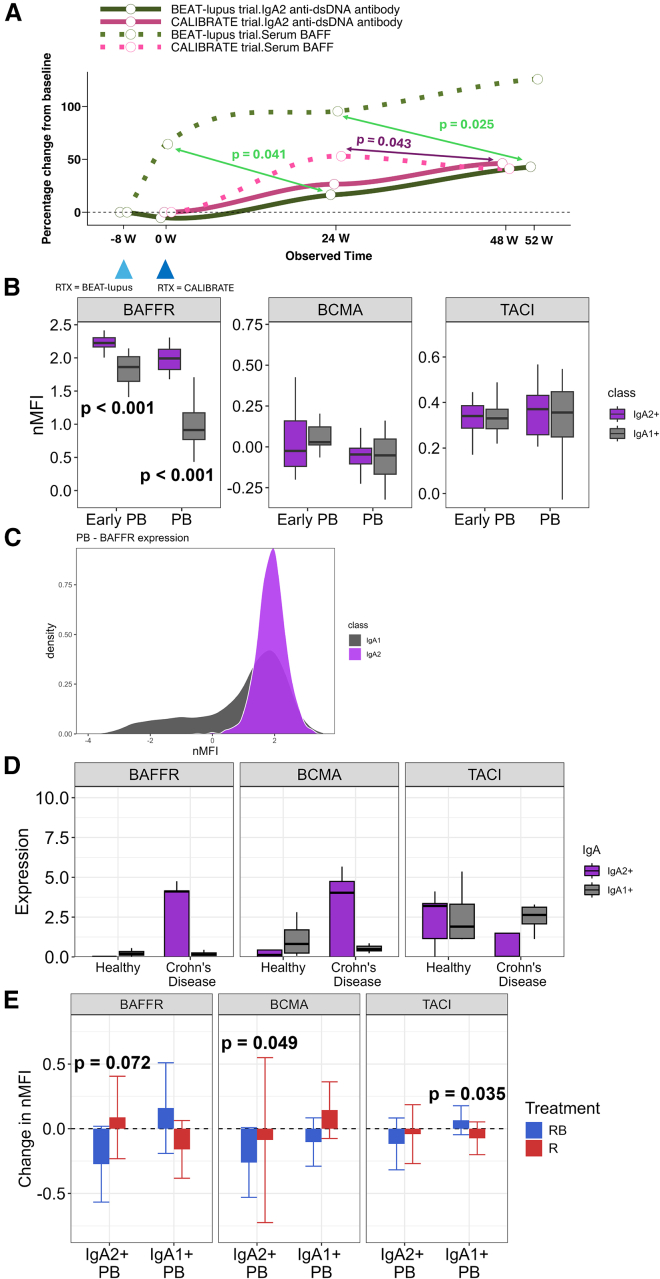
Baseline and longitudinal analysis of BAFF receptors in IgA2 compared to IgA1 plasmablasts (A) Longitudinal percentage change in serum IgA2 anti-dsDNA antibodies and BAFF levels from screening to 52 weeks in the rituximab-alone arm from both the BEAT-lupus and CALIBRATE trials (*n* = 41). The lines were smoothed using a locally estimated scatterplot smoothing (LOESS) method. A time-lagged longitudinal linear mixed-effects model was fitted, accounting for clustering by patients with random patient effects, and fixed effects of serum BAFF intersecting with trial times. Two infusions of rituximab were administered at weeks 0 and 2 (CALIBRATE) and between 4 and 8 weeks before week 0 (BEAT-lupus). The baseline samples were taken before the rituximab infusions. (B) Expression of BAFFR, BCMA, and TACI in IgA2 and IgA1 plasmablasts (cross-sectional lupus cohort, *n* = 10). Mean plus 95% confidence intervals are shown. Mann-Whitney test. (C) Histogram of BAFFR expression in IgA2 and IgA1 plasmablasts (cross-sectional lupus cohort). (D) Expression of BAFFR, BCMA, and TACI in IgA2 and IgA1 plasma cells in healthy and Crohn’s disease gut tissue from publicly available single-cell RNA sequencing data, *n* = 4 healthy controls and *n* = 5 Crohn’s. Mean plus 95% confidence intervals are shown. (E) Change in expression of BAFFR, BCMA, and TACI at week 48 compared to baseline in the CALIBRATE trial between IgA2 and IgA1 plasmablasts, *n* = 38. Mean plus 95% confidence intervals are shown. Linear mixed-effects model used for analysis. RB, belimumab after rituximab; R, rituximab. Only *p* values <0.1 are shown. See also Figure S4.

In the rituximab-alone arm of the BEAT-lupus and CALIBRATE trials, serum BAFF levels steadily increased during the trial and were associated with a subsequent rise in serum IgA2 anti-dsDNA antibody levels analyzed using a time-lagged mixed model (Figure 3A). Thus, the rise in serum BAFF at 24 weeks was associated with an increase in serum IgA2 anti-dsDNA antibody levels at 48 (CALIBRATE, *p* = 0.043) and 52 weeks (BEAT-lupus, *p* = 0.025) respectively (Figure 3A). Similarly, the rise in serum BAFF 4–8 weeks after rituximab treatment (at randomization) in the BEAT-lupus trial was associated with an increase in serum IgA2 anti-dsDNA antibody levels at 24 weeks (*p* = 0.041).

In the context of increased serum BAFF after rituximab, we sought to explore the mechanisms underpinning the differential effect upon IgA2 compared to IgA1 in response to combination belimumab after rituximab therapy compared to rituximab alone. We therefore examined the expression of BAFF receptor (BAFFR), B cell maturation antigen (BCMA), and transmembrane activator and calcium modulator and cyclophilin ligand interactor (TACI) in PBs. We performed flow cytometry with an identical panel of markers on the PBMCs from an independent cohort (10 patients with SLE analyzed) not on any biologic therapy (included in Table S2), followed by clustering analysis (Figure S4A). In this cohort, the expression of BAFFR was significantly higher in IgA2 PBs compared to IgA1 PBs (Figures 3B and 3C). No significant differences were seen in BCMA and TACI expression between the two IgA subclasses. To validate this at the RNA level, a publicly available single-cell RNA sequencing (scRNA-seq) dataset derived from the small intestine, known to be a significant reservoir of IgA PBs,^12^ of healthy individuals and individuals with Crohn’s disease was analyzed.^13^ IgA2 plasma cells displayed increased BAFFR and BCMA expression compared to IgA1 plasma cells in patients with Crohn’s but not in healthy controls (Figure 3D). To understand whether belimumab after rituximab modulates the expression of these receptors in IgA2 B cells, we looked at their expression longitudinally in the PB cluster within CALIBRATE. This revealed that there was a trend toward a reduction in BAFFR expression in IgA2 PBs after combination therapy (*p* = 0.072), whereas BAFFR expression trended upward in IgA1 PBs (Figures 3E and S4B).

### Chemokine and integrin receptor expression in IgA2 and IgA1 PBs

**Figure 4:**
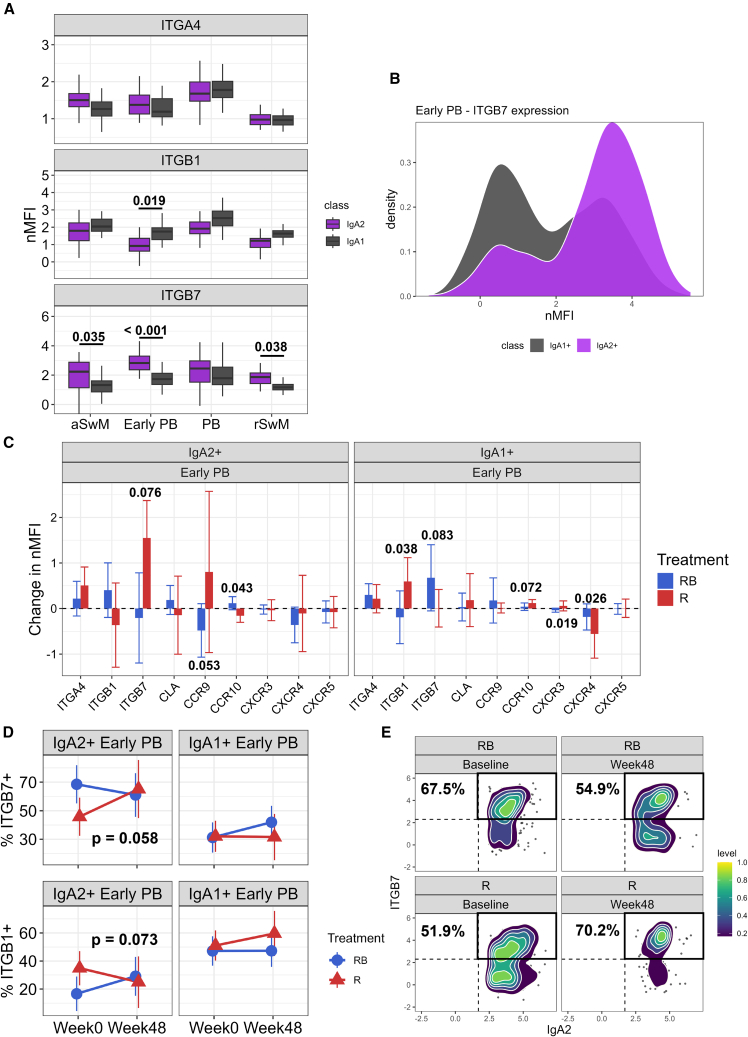
Chemokine and integrin receptor expression in IgA2 and IgA1 plasmablasts (A) Baseline nMFI of integrin expression comparing IgA2^+^ vs. IgA1^+^ cells in memory B cell and plasmablast clusters (CALIBRATE). Mean plus 95% confidence intervals are shown. Two-way ANOVA followed by Tukey HSD was used to compare integrin expression between IgA2 and IgA1 B cells. (B) Histogram of ITGB7 expression in IgA2^+^ and IgA1^+^ early plasmablasts. (C) Change in nMFI of integrin and chemokine receptors in IgA2^+^ and IgA1^+^ early plasmablasts from baseline to week 48. Linear mixed model comparing the difference in the two arms of the trial at week 48 compared to baseline. Mean plus 95% confidence intervals are shown. (D) Change in percentage of ITGB7^+^ or ITGB1^+^ in IgA2^+^ and IgA1^+^ early plasmablasts. Mean plus 95% confidence intervals are shown. Linear mixed model used to analyze difference between the two arms at 48 weeks compared to baseline (CALIBRATE). (E) Scatterplot with overlaid kernel density of ITGB7 expression, within IgA2^+^ early plasmablasts, at baseline and week 48, in both treatment arms (CALIBRATE). Black box highlights the proportion of ITGB7^+^ cells within the IgA2^+^ compartment. RB, belimumab after rituximab; R, rituximab. *n* = 38. Only *p* values <0.1 are shown. See also Figure S4.

Given the different tissue distribution of IgA1 and IgA2 B cells,^9^ we explored their chemokine and integrin receptor profile at baseline in the terminally differentiated B cell subsets. Most of the chemokine receptors assayed showed minimal differences between the two IgA subclasses (Figure S4C). However, differences were seen with respect to integrin expression (Figure 4A). IgA1 early PBs had significantly increased expression of the mucocutaneous-homing integrin ITGB1 compared to IgA2 early PBs (*p* = 0.019). Expression of ITGB7, a marker for gut homing, was significantly increased on IgA2 compared to IgA1 switched memory B cells (*p* < 0.05) and early PBs (*p* < 0.001) (Figures 4A and 4B). We next determined whether combination belimumab after rituximab affected the chemokine and integrin receptor profiles, focusing on IgA2 and IgA1 early PBs, compared to treatment with rituximab alone (Figure 4C). There was a trend toward a reduction in the expression of the gut-homing receptor CCR9 in IgA2 early PBs after combination therapy (*p* = 0.053) compared to an increased expression with rituximab alone, while ITGB7 followed the same trend (*p* = 0.076) with an increase of ITGB7 expression in the rituximab-alone arm. In contrast, IgA1 early PBs displayed the opposite pattern, with a significant reduction in ITGB1 expression after combination therapy compared to rituximab alone (*p* = 0.038), while ITGB7 expression trended upward after belimumab and rituximab therapy (*p* = 0.083). There was also a significant difference between the two arms of the trial with respect to IgA2 CCR10 (*p* = 0.043), IgA1 CXCR3 (*p* = 0.019), and IgA1 CXCR4 (*p* = 0.026). To further explore the effects of combination therapy compared to rituximab alone on ITGB7 and ITGB1, the proportions of IgA2 and IgA1 early PBs positive for these receptors were measured at baseline and week 48. The proportion of ITGB7^+^ IgA2 early PBs showed a trend toward reduction (*p* = 0.058) (Figures 4D and 4E), while the proportion of ITGB1-expressing early PBs trended upward (*p* = 0.073) (Figure 4D) after belimumab-rituximab combination therapy compared to after rituximab alone (where the proportion of ITGB7^+^ IgA2 early PBs increased). Notably, no corresponding effect was seen on IgA1^+^ early PBs.

### Effect of combination belimumab after rituximab therapy upon CD11c^+^Tbet^+^ B cells

**Figure 5:**
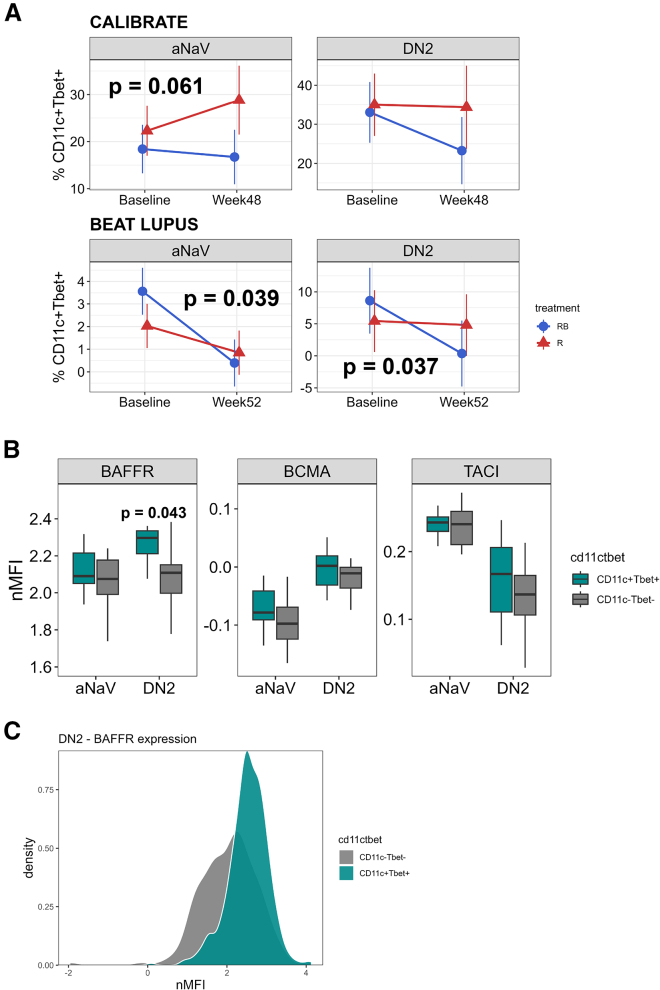
CD11c^+^Tbet^+^ B cells are targeted by belimumab after rituximab (A) Percentage of CD11c^+^Tbet^+^ B cells in activated naive (aNaV) and double-negative 2 (DN2) B cells at baseline and week 48. Mean plus 95% confidence intervals are shown. Linear mixed-effect model was used to compare values at week 48 (CALIBRATE, *n* = 38) and week 52 (BEAT-lupus, *n* = 35) adjusting for baseline values. (B) Expression of BAFFR, BCMA, and TACI in CD11c^+^Tbet^+^ compared to CD11c^−^Tbet^−^ cells in an independent cross-sectional cohort, *n* = 10. Mean plus 95% confidence intervals are shown. (C) Histogram of BAFFR expression in CD11c^+^Tbet^+^ and CD11c^−^Tbet^−^ DN2 cells. RB, belimumab after rituximab; R, rituximab. Only *p* values <0.1 are shown. See also Figure S4.

Published data on the immune effects of belimumab (without rituximab) in SLE have indicated that CD11c^+^ B cells are targeted.^14^ The majority of CD11c-high B cells in SLE co-express Tbet^15^ and are known as age-related or atypical B cells.^16^ We therefore investigated the impact of the combination therapy on those B cell clusters expressing the highest levels of CD11c and Tbet. The activated naive (aNaV) and DN2 clusters possessed the highest number of CD11c^+^Tbet^+^ B cells (Figure S4D). Visualizing the location of IgA2-positive cells overlaid on the uniform manifold approximation and projection (UMAP) of B cell subsets indicated that there were fewer IgA2-positive cells in the atypical B cell clusters compared to their conventional counterparts (Figure 2D). We measured the proportion of CD11c^+^Tbet^+^ cells in these two B cell clusters after treatment. There was a trend toward a reduction within the aNaV compartment in the combination arm compared to rituximab alone at week 48 in the CALIBRATE trial (*p* = 0.061) and a significant reduction in aNaV (*p* = 0.039) and DN2 (*p* = 0.037) in the combination arm of the BEAT-lupus trial compared to rituximab alone at week 52 compared to baseline (Figure 5A). We checked BAFFR expression in CD11c^+^Tbet^+^ B cells from patients with lupus to determine whether this could account for their increased reduction following belimumab-rituximab combination therapy paralleling the observations with IgA2 B cells. Using an independent cross-sectional cohort, CD11c^+^Tbet^+^ B cells expressed higher BAFFR expression compared to CD11c^−^Tbet^−^ cells (*p* = 0.043) (Figures 5B and 5C).

### The B cell subsets targeted by belimumab after rituximab produced more IL-10

**Figure 6:**
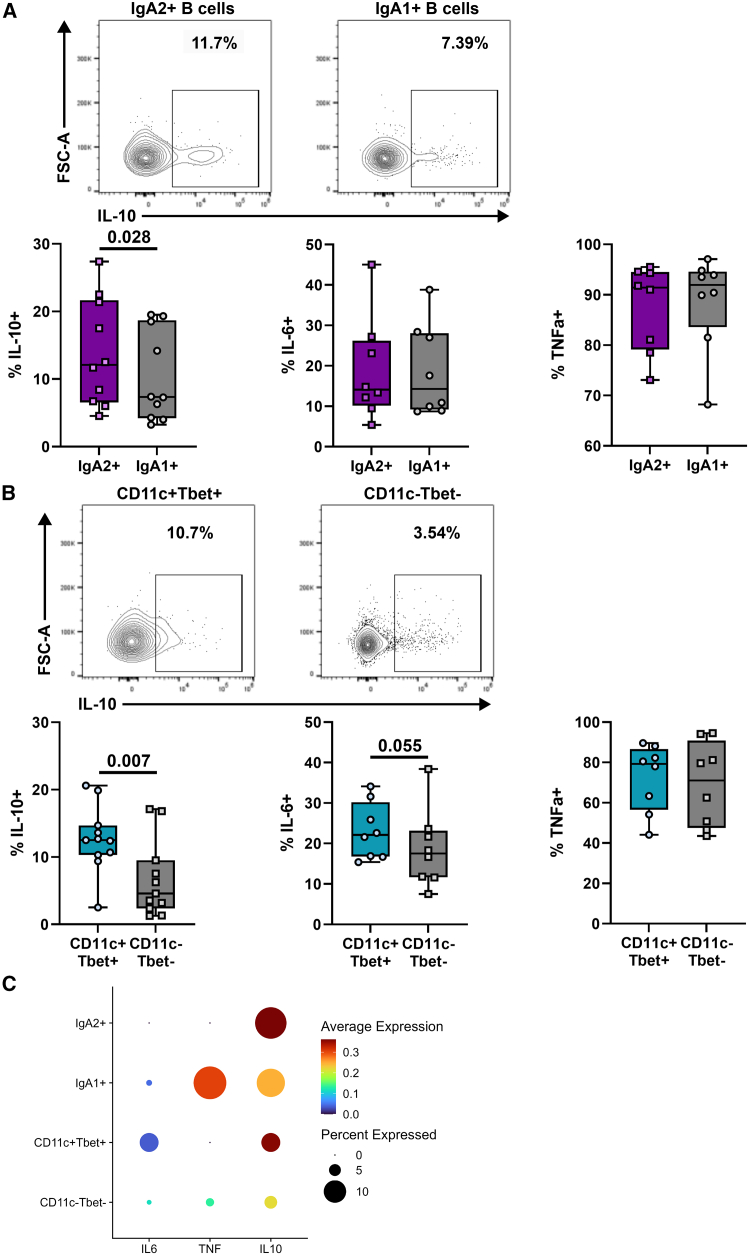
IgA2 and CD11c^+^Tbet^+^ B cell cytokine production in patients with SLE (A and B) Summary boxplots of IL-10, IL-6, and TNF-α expressing (A) IgA2 vs. IgA1 and (B) CD11c^+^TBet^+^ vs. CD11c^−^TBet^−^ B cells stimulated with CpG for 48 h. Representative fluorescence-activated cell sorting plots of IL-10 expression are shown. Mann-Whitney was used for analysis. *n* = 9. Mean plus 95% confidence intervals are shown for (A) and (B). (C) Expression and percent positivity of IL-6, TNF, and IL-10 genes in IgA2^+^, IgA1^+^, CD11c^+^Tbet^+^, and CD11c^−^Tbet^−^ B cells from a publicly available single-cell RNA sequencing dataset derived from purified human B cells stimulated with CpG. Only *p* values <0.1 are shown.

We next measured the production of interleukin (IL)-10, IL-6, and tumor necrosis factor alpha (TNF-α) from IgA2 and CD11c^+^Tbet^+^ B cells isolated from 9 patients with SLE (independent cohort, Table S2). Total IgA2 B cells, stimulated with CpG for 48 h, produced significantly higher levels of IL-10 compared to IgA1 B cells (Figure 6A), while no significant difference was observed in IL-6 and TNF-α production from B cells stratified by IgA subclass. Total CD11c^+^Tbet^+^ B cells produced more IL-10 than their CD11c^−^Tbet^−^ counterparts (Figure 6B). To confirm these differences in IL-10 production, we analyzed IL-10 RNA expression in a publicly available scRNA-seq dataset derived from B cells stimulated with CpG.^17^ There was greater expression of IL-10 and a higher proportion of IL-10-positive cells within IgA2 B cells and CD11c^+^Tbet^+^ B cells compared to IgA1 B cells and CD11c^−^Tbet^−^ B cells, respectively (Figure 6C). Expression of TNF and IL-6 are also shown for comparison.

## Discussion

Rituximab remains a frequently utilized advanced therapy for lupus, particularly lupus nephritis, in accordance with established guidelines.^18^ In one study, SLE was the commonest diagnosis for off-label use of rituximab.^19^ Its popularity in publicly funded health systems is partly attributable to its off-patent status and the low cost of biosimilars. However, following rituximab therapy in patients with SLE, BAFF levels increase and are associated with worsening disease, limiting its effectiveness as a therapy.^4^ The data presented here suggest that IgA2 PBs and, to a lesser extent, CD11c^+^Tbet^+^ B cells are two distinct terminally differentiated effector B cell lineages present in patients with lupus that are particularly sensitive to the rise in BAFF levels following rituximab due to their increased BAFFR expression. Both the CALIBRATE and BEAT-lupus trials indicate that treating patients with belimumab after rituximab led to a significant decline in peripheral blood IgA2-expressing PBs and aSwM B cells, cells which likely participate in the ongoing autoimmune response in SLE, compared to treatment with rituximab alone. Of greatest clinical utility, those patients with elevated serum IgA2 anti-dsDNA antibodies at baseline were almost 17 times more likely to respond to belimumab after rituximab compared to rituximab alone based on the combined data from the CALIBRATE and BEAT-lupus trials. This validation of IgA2 anti-dsDNA antibodies as a theragnostic biomarker for combination belimumab after rituximab from two independent clinical trials offers the prospect of adopting a precision medicine approach for those patients with high levels of this biomarker who are treated with rituximab.

Although IgA1 is the predominant IgA subclass in the serum, IgA1 anti-DNA antibodies were not significantly elevated compared to healthy controls in the CALIBRATE trial. In contrast, serum IgA2 anti-dsDNA antibody levels were raised at baseline compared to healthy controls and were reduced by belimumab after rituximab compared to rituximab alone. Of relevance, previous data from analysis of the BEAT-lupus trial suggested that IgA2 anti-dsDNA antibodies were associated with renal disease^7^ consistent with the serological results reported here from the CALIBRATE trial, which recruited patients with active lupus nephritis. The contrasting results obtained between the two IgA subclasses are likely due to the increased BAFFR expression in IgA2 compared to IgA1 B cells. We documented increased BAFFR expression in IgA2 B cells not only in SLE but also in the inflamed gut mucosa of Crohn’s disease. These data not only support the hypothesis that BAFF predominantly drives IgA2 antibody production through promoting class switching to this subclass^20^ but also provides mechanistic insight into why IgA2 anti-dsDNA antibody levels are a biomarker for response to belimumab after rituximab in the context of rising BAFF levels. Taken together, we hypothesize that the rise in BAFF following rituximab in BEAT-lupus and CALIBRATE could trigger a wave of autoreactive IgA2 production, for instance, in the gut where IgA2 is the dominant IgA subclass. The ensuing lupus flare would be markedly sensitive to BAFF blockade. Elevated IgA2 anti-DNA antibody levels before rituximab is administered may be a biomarker of a lupus endotype sensitive to a surge in BAFF following rituximab. BAFF blockade may be less effective in diseases driven by IgA1 autoantibodies, and indeed, there are no reports in the literature of belimumab’s efficacy in IgA nephropathy, which is thought to be principally associated with IgA1 autoantibodies.^21^

The integrin expression of IgA2 B cells is consistent with their predominance at mucosal surfaces such as in the gut. Expression of ITGA4 and ITGB7 receptors (which dimerize to form the integrin α4β7) characterized the IgA2 compartment and was reduced on IgA2 PBs after combination belimumab after rituximab therapy. A recent study demonstrated that the α4β7 integrin can mediate adhesion and function of innate lymphoid cells in the kidney,^22^ raising the possibility of a migration pathway of IgA2 PBs from the gut to the kidney in lupus nephritis that is targeted by belimumab after rituximab therapy. The targeting of IgA2 B cells by belimumab after rituximab and their predominance in the gut may suggest that the main site of action for belimumab is in the gut or associated lymphoid structures. It is possible that other IgA2 specificities against host or microbial antigens could also be targeted. There is some suggestion in the literature that IgA1 tends to bind to protein antigens, whereas IgA2 binds to non-protein antigens such as polysaccharides.^23^ This combination of biologic therapies has been trialed for other conditions such as Sjogren’s syndrome,^24^ but it is unknown whether there are any disease-related antigen specificities bound by IgA2 autoantibodies that could be used to predict response.

IgA2 production predominated within the switched memory and PB phenotypes, whereas IgA1 expression appeared to be more evenly distributed among B cell subsets. This is in line with a previous scRNA-seq study that found that the majority of double-negative B cell clusters expressed IgA1, but only few cells expressed IgA2.^25^ In addition to IgA2 PBs, CD11c^+^Tbet^+^ B cells had increased BAFFR expression and were targeted by belimumab-rituximab therapy. B cells expressing CD11c and Tbet, sometimes referred to as atypical or age-related B cells, have been associated with SLE pathogenesis.^26^^,^^27^ IgA2 PBs and the CD11c^+^Tbet^+^ B cells produced more IL-10 in response to stimulation compared to IgA1 and CD11c^−^Tbet^−^ B cells, respectively. IL-10 promotes the differentiation and proliferation of autoreactive B cells into plasma cells, potentially exacerbating autoantibody-driven inflammation in SLE.^28^^,^^29^ Moreover, BAFF signaling has been reported to promote IL-10 production and IgA2 production.^30^ Serum IL-10 levels are reduced in patients with SLE treated with belimumab.^31^

Our data suggest that the cautionary advice regarding the combination therapy of belimumab and rituximab for SLE issued by regulatory authorities should be reconsidered for patients who are biomarker positive. Indeed, this caution arose from a trial of patients with lupus where fewer patients were taking immunosuppressants and steroids at baseline,^32^ compared to the participants recruited to BEAT-lupus and CALIBRATE, both of which recruited patients with disease refractory to conventional therapy. Moreover, in both the BEAT-lupus and CALIBRATE trials, there were numerically more infection-related serious adverse events in the rituximab-alone group compared to belimumab followed by rituximab.^6^^,^^11^

In conclusion, baseline serum IgA2 anti-dsDNA antibody levels were strongly associated with response to belimumab after rituximab therapy in two independent clinical trials in SLE. These results provide not only validation but also mechanistic insights that will facilitate the progression of this biomarker toward routine clinical practice. The biomarker relies on a simple technology (ELISA), which is already in use in hospital laboratories, and thus could be easily incorporated into routine care as a companion theragnostic biomarker. We propose that for those patients in whom rituximab is considered appropriate to treat disease that has been refractory to conventional therapy, patients would be stratified into low and high serum IgA2 anti-dsDNA antibody levels, and those with high levels would also receive belimumab soon after the rituximab. Patients with low IgA2 anti-dsDNA antibody levels would continue with rituximab without belimumab as is current standard practice in the UK and some other countries. To prospectively test the validity of the biomarker and build a sufficiently robust case to facilitate approval of this personalized precision medicine approach by healthcare providers, we propose a biomarker enrichment trial, where only patients with disease refractory to conventional therapy and with high IgA2 anti-dsDNA antibody levels are recruited and randomized to either belimumab after rituximab or rituximab alone. Because of the substantial effect size predicted for biomarker-positive patients (58% point difference between combination and rituximab alone, see Figure 2B), far fewer patients will be needed to achieve sufficient power to validate the biomarker using a biomarker enrichment strategy compared to a conventional clinical trial (14% point difference between the two arms for all participants irrespective of the biomarker). This approach underscores the substantial value of biomarker enrichment in clinical trials, enabling the adoption of a precision medicine approach for patients with lupus.

### Limitations of the study

There were few non-responders in either arm of the CALIBRATE trial compared to the BEAT-lupus trial, making it more difficult to achieve statistical significance in relation to clinical response and the association with predictive biomarkers. Nevertheless, the serum IgA2 anti-dsDNA baseline data from CALIBRATE as a predictor of response are identical in direction to the comparable data from BEAT-lupus, and the combined OR for response prediction derived from both trials was substantial. We compared cumulative corticosteroid use in the CALIBRATE and BEAT-lupus trial and found that participants in the former trial were exposed to substantially more steroids during the trial compared to BEAT-lupus, providing a potential explanation for the few non-responders in the CALIBRATE trial. These data were collected retrospectively, highlighting the need for a prospective clinical trial to test the value of IgA2 anti-dsDNA antibodies as a predictive biomarker. Both BEAT-lupus and CALIBRATE were designed to reflect real-world practice and specifically enrolled participants with severe disease refractory to conventional therapy who may have been excluded from other lupus trials. Nevertheless, there were exclusion criteria for both trials, and some patients with the worst disease may not have been included. Finally, we only had access to peripheral blood to measure the changes in IgA2-producing B cells, limiting insights into changes in tissues such as the large bowel where IgA2 predominates.

## Resource availability

### Lead contact

Requests for further information, resources, and reagents should be directed to the lead contact, Michael R. Ehrenstein (m.ehrenstein@ucl.ac.uk).

### Materials availability

This study did not generate new unique reagents.

### Data and code availability

• The spectral flow cytometry data reported in this study have been deposited in the UCL Research Data Repository (DOI: 10.5522/04/29099729). Human scRNA-seq data deposited at ArrayExpress https://www.ebi.ac.uk/biostudies/arrayexpress/studies/E-MTAB-13872 and https://www.gutcellatlas.org were also analyzed. Requests for additional data must include a detailed description of the research objectives and will be considered for non-commercial, research purposes only. To protect participant privacy, no personally identifiable or sensitive clinical information will be provided. All requests must comply with the consent agreements established with study participants.
• Code for the computational analysis of spectral flow cytometry data is available as listed in the key resources table (DOI: https://doi.org/10.5281/zenodo.15746523).
• Any additional information required to reanalyze the data reported in this paper is available from the lead contact upon request.

## Acknowledgments

The authors gratefully acknowledge the CALIBRATE and BEAT-lupus study teams. We are indebted to the patient participants in the CALIBRATE and BEAT-lupus trials and other patients who contributed samples. We thank Ellie Hawkins and Linda Tomson for recruiting patients and collecting the samples at University College London Hospitals NHS Foundation and Jamie Evans (the Rayne Flow Cytometry Facility, UCL) for flow cytometry technical support. We thank Dr. Thomas McDonnell for advice about ELISA design and Mariea Parvaz for performing the flow cytometry on the samples from the BEAT-lupus trial. M.R.E. is partly funded through a block grant from the National Institutes of Health Research to University College London Hospitals Biomedical Research Centre. This research was supported by 10.13039/501100012041Versus Arthritis (grant no. 22961) to M.R.E. and M.R.A.S.

## Author contributions

D.M., M.R.A.S., and M.R.E. designed the study. D.M. and M.R.A.S. acquired, analyzed, and had access to the data. J.A.J. provided CALIBRATE serum BAFF data. K.C. analyzed the data and provided additional statistical support. D.M., M.R.A.S., and M.R.E. wrote the manuscript with additional review and critique provided by L.A.C., K.C., and J.A.J. All authors reviewed the manuscript and approved the final version.

## Declaration of interests

M.R.E. has received grant/research support from GSK. M.R.E. and J.A.J. have received consultancy fees for attending GSK advisory boards. M.R.A.S. and M.R.E. are named on patent application no. 18/864,514 (IgA2 anti-dsDNA antibodies as a biomarker in SLE, the patent is to University College London).

## STAR★Methods

### Key resources table

**Table:** 

REAGENT or RESOURCE	SOURCE	IDENTIFIER
**Antibodies**		

FACS: CD3 (UCHT1)	Biolegend	Cat# 300431;RRID:AB_1595437
FACS: CD19 (SJ25C1)	BD Biosciences	Cat# 563549;RRID:AB_2738272
FACS: IgD (IA6-2)	Biolegend	Cat# 348242;RRID:AB_2629809
FACS: CD27 (M-T271)	Biolegend	Cat# 356420;RRID:AB_2562603
FACS: CD38 (HB7)	BD Biosciences	Cat# 612824;RRID:AB_2870148
FACS: CD21 (HB5)	eBioscience	Cat# 46-0219-42;RRID:AB_2573672
FACS: CD24 (ML5)	BD Biosciences	Cat# 741607;RRID:AB_2871015
FACS: CD95 (DX2)	Biolegend	Cat# 987102;RRID:AB_3068068
FACS: CXCR3 (G025H7)	Biolegend	Cat# 353762;RRID:AB_2904376
FACS: CXCR4 (12G5)	Biolegend	Cat# 306508;RRID:AB_314614
FACS: CXCR5 (J252D4)	Biolegend	Cat# 356916;RRID:AB_2562290
FACS: CLA (KPL-1)	BD Biosciences	Cat# 746886;RRID:AB_2871685
FACS: CCR9 (C9Mab-1)	BD Biosciences	Cat# 752592;RRID:AB_2917579
FACS: CCR10 (1B5)	BD Biosciences	Cat# 564772;RRID:AB_2738944
FACS: ITGA4 (9F10)	BD Biosciences	Cat# 749454;RRID:AB_2873822
FACS: ITGB1 (TS2/16)	Biolegend	Cat# 303026;RRID:AB_2716098
FACS: ITGB7 (FIB504)	BD Biosciences	Cat# 751567;RRID:AB_2875562
FACS: CD11c (B-ly6)	BD Biosciences	Cat# 563929;RRID:AB_2744276
FACS: Tbet (4B10)	Biolegend	Cat# 644828;RRID:AB_2565677
FACS: BAFFR (11C1)	BD Biosciences	Cat# 749944;RRID:AB_2874179
FACS: BCMA (19F2)	Biolegend	Cat# 357516;RRID:AB_2686991
FACS: TACI (1A1-K21-M22)	BD Biosciences	Cat# 744147;RRID:AB_2742033
FACS: IgA1 (B3506B4)	Southern Biotech	Cat# 9130-02;RRID:AB_2796652
FACS: IgA2 (A9604D2)	Southern Biotech	Cat# 9140-31;RRID:AB_2796666
FACS: IgG (G18-145)	BD Biosciences	Cat# 741396;RRID:AB_2870890
FACS: IL-6 (MQ2-13A5)	BD Biosciences	Cat# 563279;RRID:AB_2738113
FACS: IL-10 (JES3-19F1)	Biolegend	Cat# 506804;RRID:AB_315454
FACS: TNFa (Mab11)	Biolegend	Cat# 502929;RRID:AB_2204080
ELISA: IgA1-HRP	Cambridge Bioscience	Cat# 9130-05;RRID:AB_2796654
ELISA: IgA2-HRP	Cambridge Bioscience	Cat# 9140-05;RRID:AB_2796662

**Biological samples**		

CALIBRATE trial PBMCs	Atisha-Fregoso et al.^11^	N/A
CALIBRATE trial serum	Atisha-Fregoso et al.^11^	N/A
BEAT-lupus trial PBMCs	Shipa et al.^7^	N/A
UCL cross-sectional SLE PBMCs	This paper	N/A

**Chemicals, peptides, and recombinant proteins**		

Protamine sulfate	Sigma-Aldrich	Cat# P4505
Calf thymus DNA	Cambridge Bioscience	HY-109517
CpG ODN 2395	Miltenyi Biotec	130-100-283

**Deposited data**		

Flow cytometry FCS files	This paper	DOI: 10.5522/04/29099729
Small intestine scRNA-seq data	James et al.^13^	gutcellatlas.org
Healthy control B cells scRNA-seq	Bradford et al.^17^	https://www.ebi.ac.uk/biostudies/arrayexpress/studies/E-MTAB-13872

**Software and algorithms**		

Flowjo v10	BD Biosciences	https://www.flowjo.com/
R 4.3.3	N/A	https://cran.r-project.org/
CATALYST 1.26.0	Nowicka et al.^33^	https://github.com/HelenaLC/CATALYST
Harmony 1.2.0	Korsunsky et al.^34^	https://github.com/immunogenomics/harmony
Slingshot 2.7.0	Street et al.^35^	https://github.com/kstreet13/slingshot
Seurat 5.0.2	Hao et al.^36^	https://github.com/satijalab/seurat
Lme4 1.1–35.1	Bates et al.^37^	https://github.com/lme4/lme4
Code for spectral flow cytometry analysis	This paper	https://doi.org/10.5281/zenodo.15746523

### Experimental models and study participant details

We analyzed peripheral blood mononuclear cells (PBMC) and serum samples from patients recruited to the previously published CALIBRATE trial^11^ (NCT02260934). Briefly, CALIBRATE was a phase II multicentre, randomized, controlled, open-label trial of cyclophosphamide plus rituximab followed by randomization to belimumab plus standard of care or standard of care alone for 48 weeks in patients with active lupus nephritis. 38 participants out of the 43 enrolled to the CALIBRATE trial had PBMCs available at baseline and 48-week timepoints. Participant information can be found in Table S1. Response was defined as complete or partial renal response.^11^ We also analyzed PBMC from 35 participants in the BEAT-lupus trial (ISRCTN 47873003) at baseline and the 52-week time point; clinical response was defined as major clinical response.^7^ Major clinical response (MCR) was defined as reduction in BILAG–2004 (British Isles lupus assessment group-2004) index A/B scores to BILAG–2004 C/D or remain E in all domains, a reduction in steroid dose to ≤7.5mg daily and a modified SLEDAI (Systemic lupus erythematosus disease activity index 2000) –2K score ≤2 (without including the anti-dsDNA antibody component). A cross-sectional cohort of SLE patients was also studied for some experiments, see Table S2.

The Hampstead Research Ethics Committee-London approved the analysis of the participant samples (ref. 13/LO/0999). The study was conducted in accordance with the principles of the Declaration of Helsinki Good Clinical Practice guidelines. All patients provided written informed consent before enrollment.

### Method details

#### Flow cytometry

For both conventional and spectral flow cytometry, PBMCs were stained using a panel of antibodies. All surface staining was performed for 30 min at 4°C. Samples were fixed and permeabilized overnight at 4°C. Intracellular staining was performed for 40 min at 4°C. The list of antibodies used, along with their concentrations and clones, are included in The Key Resources Table. All antibodies were used at 1:100 concentration except for CD11c, BCMA, TACI, IgA1, IgA2, IgG, IL-6, IL-10 and TNFa (all 1:50) and Tbet (1:33). All antibodies were used in the spectral flow cytometry panel for the CALIBRATE samples, whereas the following panel was used to analyze the BEAT LUPUS samples: (CD19,CD24,CD27,CD38,IgD,CD21,Tbet,CD11c,CD95,CXCR3) using conventional flow cytometry. Spectral acquisition was performed on a Sony ID7000 machine; conventional flow cytometry was performed on a BD Fortessa. Initial pre-processing of the spectral data was performed on FlowJo (version 10) before transfer to R, whilst all conventional analysis was performed using FlowJo.

#### ELISA

In-house ELISAs were used to measure IgA1 and IgA2 anti-dsDNA antibodies.^7^ Plates were precoated with 100ul/well protamine sulfate (Sigma) for 45 min at 4°C and then, after washing, coated overnight at room temperature with 10ug/ml calf thymus dsDNA (Cambridge Bioscience). Serum samples were added at a 1:32 dilution and incubated for 2 h at room temperature. After washing, bound antibody subclasses were detected using HRP-conjugated anti-human IgA1 or IgA2 (Cambridge Bioscience). Results are reported in arbitrary units, which are relative OD values (OD at 450nm) compared to the same positive control sample, which was serially diluted in each plate to produce a standard curve (Figure S5A). The reproducibility of the ELISA was tested using the same samples tested at different times and by different operators (Figure S5B). Serum concentrations of BAFF/BLyS were analyzed by xMAP multiplex assays (Affymetrix, Santa Clara, CA) as previously described^37^ (CALIBRATE) or by ELISA (R&D Systems, cat # DY124-05) (BEAT-lupus).

#### CpG stimulation

PBMCs were seeded in a 24 well plate at a density of 2x10∧6 cells/ml in RPMI and cultured for 48 h at 37°C in the presence of 1uM CpG. PMA/Ionomycin was added to all wells for the last 4.5 h before harvesting. PBMCs were then stained for cytokine expression (IL-10, TNFα and IL-6) using spectral flow cytometry as described above.

### Computational analysis of spectral flow cytometry data

#### Processing

FCS files were exported after acquisition and loaded into Flowjo. CD3^−^CD19^+^ live single B cells were selected and exported as FCS files. The FCS files were loaded into R (Version 4.3.3) and processed using the CATALYST package (version 1.26.0).^38^ The data was converted into a SingleCellExperiment object. Expression was normalized using the CATALYST “prepData” function, with the cofactor parameter set to 1000. To correct for batch effects, the Harmony package (version 1.2.0) was utilized.^33^ The HarmonyMatrix function was used, with batch, age and gender set as covariates.

#### Clustering

Clustering was performed using CATALYST’s cluster function. The maximum k value was set to 30 to allow inspection of a wide range of clustering resolutions. For visualization of the UMAP, samples were randomly downsampled to 1000 cells when running the “runDR” function due to computational constraints.

#### Pseudotime analysis

The Slingshot package (version 2.7.0) was used to perform pseudotime analysis.^34^ The resting naive/transitional cluster was set as the origin point for the trajectories.

#### Analysis of public single-cell RNA seq data

For analysis of BAFFR (and BCMA and TACI), processed data derived from B cells was downloaded from gutcellatlas.org and loaded into R. Using Seurat^35^ (version 5.02) the IgA plasmablast cluster (previously identified by the original authors^13^) was extracted and *IGHA1* and *IGHA2* gene expression inspected to determine cutoffs for IgA1 and IgA2 plasmablasts. Expression of the BAFFR, BCMA and TACI genes was then measured between IgA1 and IgA2 plasmablasts, categorized into healthy controls and patients with Crohn’s disease.

For analysis of IL10 (and IL6 and TNF) gene expression on healthy control B cells from Bradford et al.,^17^ the raw barcode, feature and matrix files were downloaded from ArrayExpress (E-MTAB-13872) and processed using Seurat. IgA1+, IgA2+, CD11c+Tbet+ and CD11c-Tbet- B cells were selected using visually determined cutoffs of the *IGHA1*, *IGHA2*, *ITGAX* and *TBX21* genes. *IL10*, *IL6* and *TNF* were then inspected and plotted using the DotPlot function.

### Quantification and statistical analysis

For all single timepoint analysis, the default Mann Whitney test built into R was used except where stated. For longitudinal analysis, a linear mixed effects model was used from the lme4 package^36^ to estimate the mean change from baseline to the week 48 timepoint. Here, age and gender were covariates used in the model, along with the specification of patient ID as the random effect, to account for the paired nature of the data. *p* values for figures using the linear mixed effects model represent the difference between the week 48/52 values considering the baseline values, as well as covariates. For the time-lagged analysis, we applied a longitudinal linear mixed-effects model to assess whether changes in serum BAFF levels at earlier time points were associated with subsequent changes in serum IgA2 anti-dsDNA antibody levels. Random patient effects were included to account for clustering, and fixed effects for serum BAFF were incorporated, interacting with trial time points to capture the delayed association between BAFF and IgA2.

To determine the optimal cut-point of IgA2 anti-dsDNA levels assayed by ELISA in predicting treatment response, we applied the Youden method which maximises the sum of sensitivity and specificity calculated from the receiver operating characteristic curve. We also carried out logistic regression analyses to obtain odds ratios to identify a threshold of IgA2 anti-dsDNA level at which there is greatest discrimination in treatment effect. Details of measurements and n number (where n = number of patients) can be found in the figure legends.

#### Additional resources

The CALIBRATE clinical trial was conducted by the Immune Tolerance Network and sponsored by the National Institute of Allergy and Infectious Diseases (NIAID) under award UM1AI109565^11^ (https://clinicaltrials.gov/study/NCT02260934). The BEAT-lupus trial (https://www.isrctn.com/ISRCTN47873003) was sponsored by University College London and funded by VersusArthritis under award number 20873 and GSK.^6^

## Supplemental information

Document S1. Figures S1–S5 and Tables S1 and S2

Document S2. Article plus supplemental information
