# Supplementary material for: IgA2+ B cells and IgA2 anti-dsDNA antibodies are selectively targeted by belimumab after rituximab therapy in systemic lupus erythematosus
Source: Cell Rep Med. 2025 Jul 23;6(8):102247. doi: 10.1016/j.xcrm.2025.102247 (PMC12432362; doi:10.1016/j.xcrm.2025.102247)
Supplement: Document S1. Figures S1–S5 and Tables S1 and S2 [file mmc1.pdf]

Cell Reports Medicine, Volume *6*

Supplemental information

IgA2$^+$ B cells and IgA2 anti-dsDNA antibodies are selectively targeted by belimumab after rituximab therapy in systemic lupus erythematosus

Daniel McCluskey, Muhammad R.A. Shipa, Kashfia Chowdhury, Judith A. James, Laura A. Cooney, and Michael R. Ehrenstein

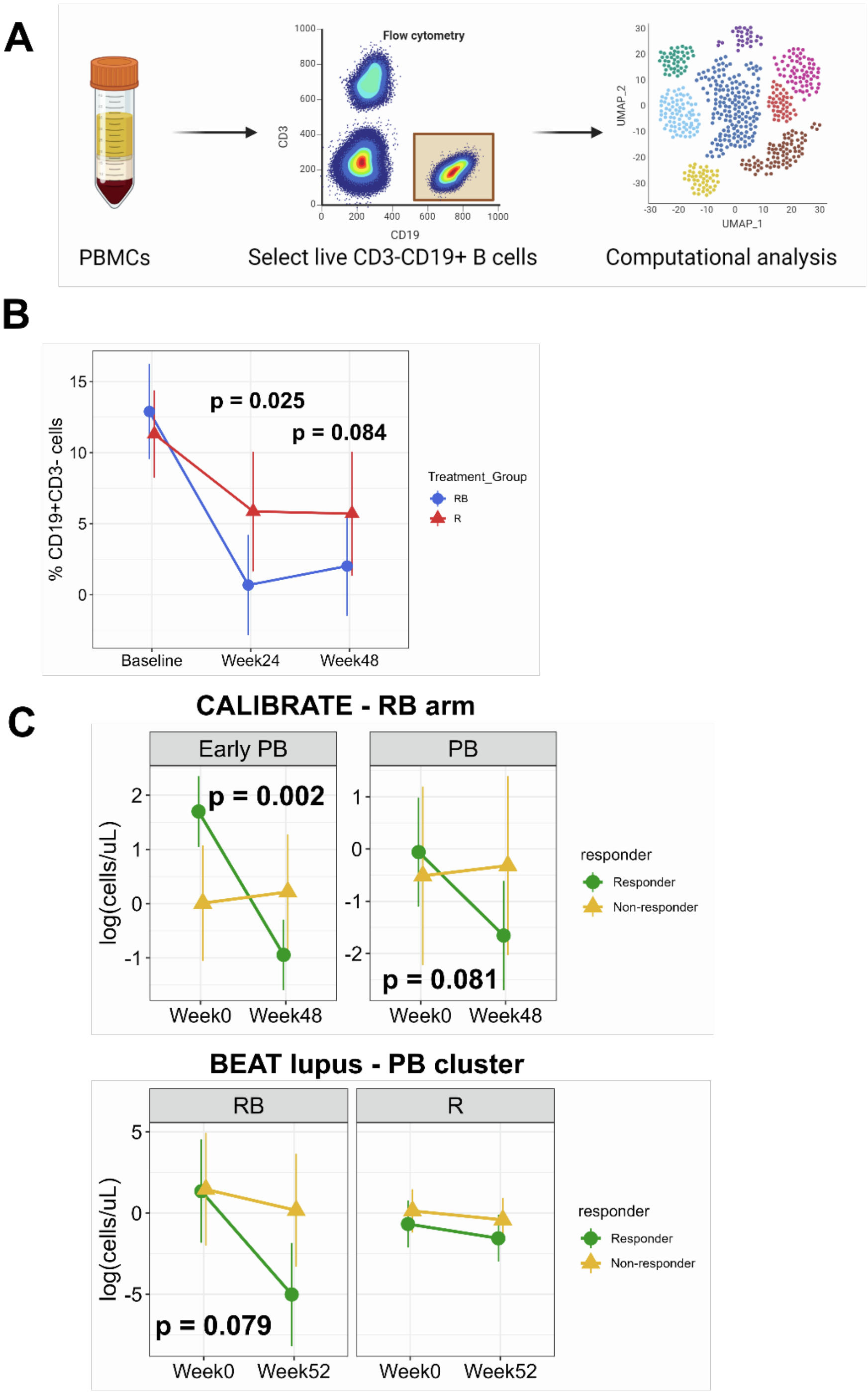

Supplementary Figure 1. Changes in B cell and plasmablast numbers in CALIBRATE and BEAT-lupus, related to Figure 1

A) Schematic of experimental design. B) Proportion of CD19+ B cells determined by conventional gating at different timepoints in the CALIBRATE trial (n=38). Mean plus 95% confidence intervals are shown. Linear mixed model comparing the difference in the two arms of the trial at week 24 and week 48 compared to baseline. C) Change in absolute counts of plasmablast populations stratified by clinical response status. The CALIBRATE trial lacked information on responder status in the rituximab alone arm. CALIBRATE combination (RB) arm: 11 responders, 5 non-responders; BEAT-lupus combination (RB) arm: 12 responders, 5 non-responders; BEAT-lupus rituximab (R) alone arm: 8 responders, 7 non-responders with matching PBMC and clinical data. Mean plus 95% confidence intervals are shown. Linear mixed model used to calculate p values. RB = belimumab after rituximab, R = rituximab. Only P values < 0.1 are shown.

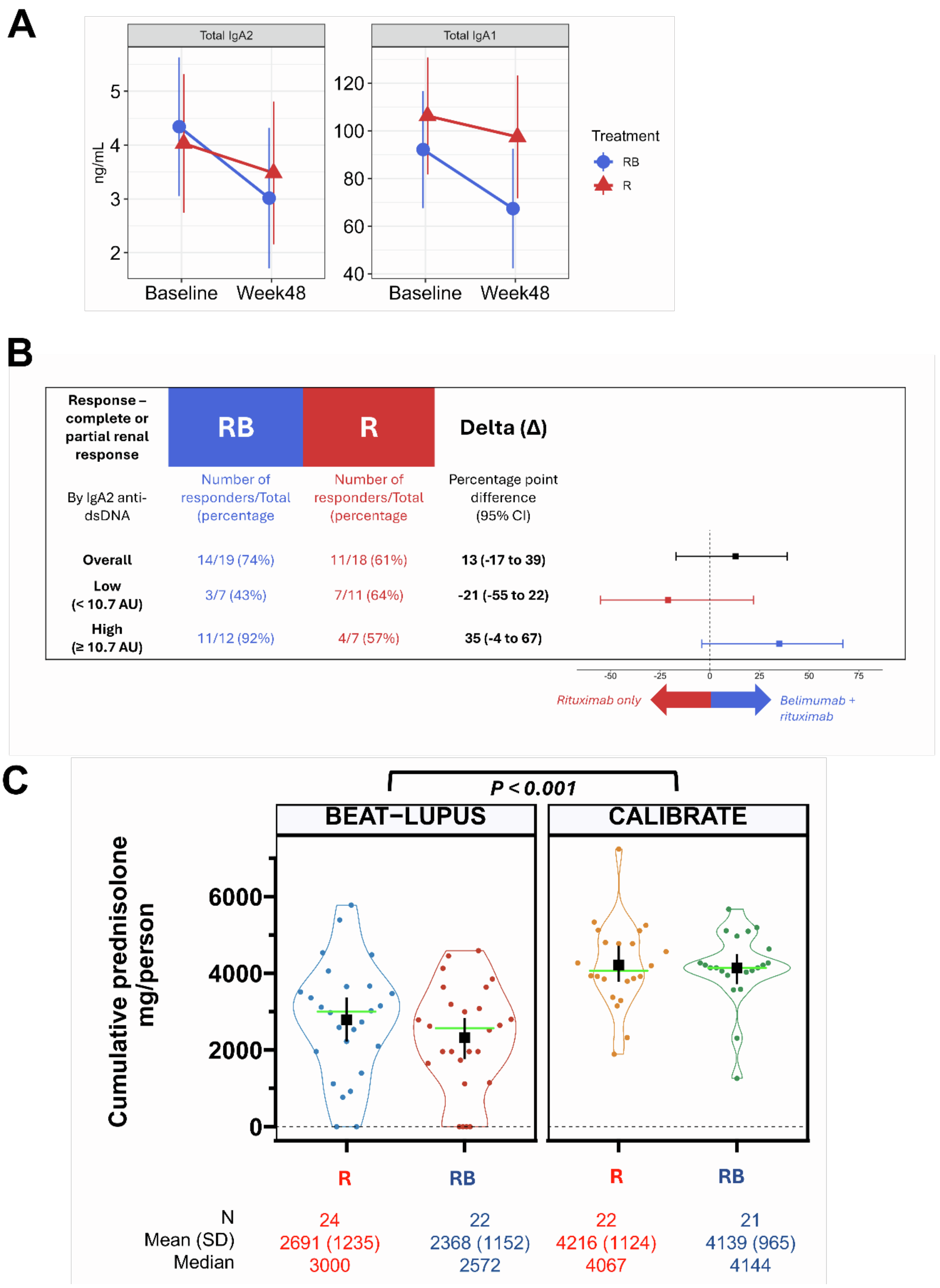

Supplementary Figure 2. Longitudinal changes in serum IgA2 and IgA1, baseline IgA2 anti-dsDNA antibody as an effect modifier of response, cumulative prednisolone in the BEAT-lupus and CALIBRATE trial, related to Figure 2

A) Serum total IgA2 and IgA1 at baseline and week 48 measured by ELISA (CALIBRATE). Mean plus 95% confidence intervals are shown. Linear mixed model comparing the difference in the two arms of the trial at week 48 compared to baseline. N = 37. Values are the mean of two technical replicates. B) Baseline serum IgA2 anti-dsDNA antibody levels were categorized into high (≥10.7 AU) or low (<10.7 AU) groups as an effect modifier of clinical response (complete or partial renal response) at 48 weeks (CALIBRATE). C) Cumulative dose of prednisolone (mg/person) in each treatment arm in the BEAT-LUPUS (through to 52 weeks) and CALIBRATE (through to 48 weeks) trials. Black boxes and lines indicate mean values with 95% confidence intervals, green horizontal lines denote median values. P value was derived from the Mann-Whitney test. RB = belimumab after rituximab, R = rituximab. Only P values < 0.1 are shown.

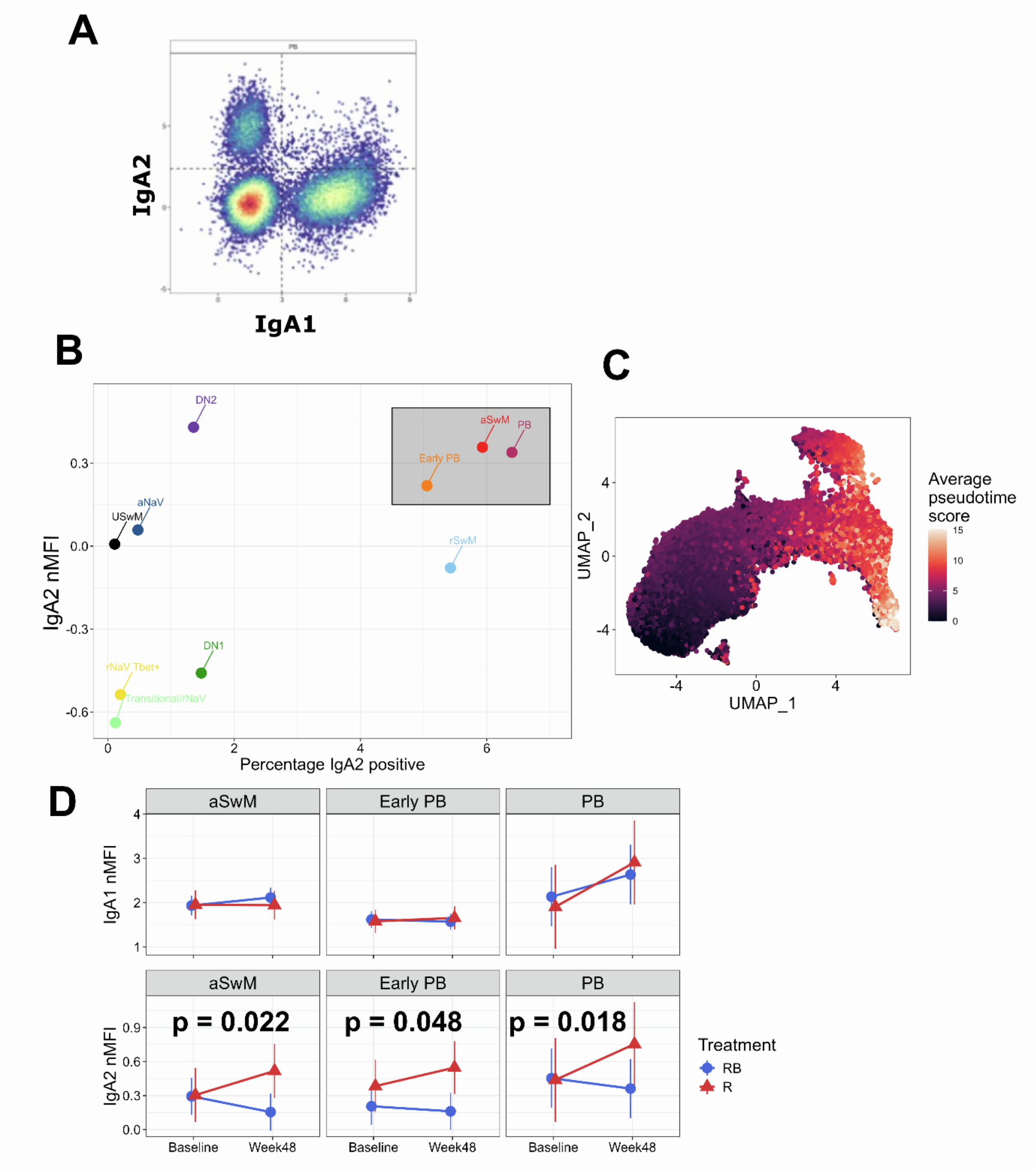

Supplementary Figure 3. IgA2 and IgA1 expression in B cell subsets, related to Figure 2

A) Scatterplot of IgA2 and IgA1 expression in the plasmablast cluster (CALIBRATE), coloured by density. Dotted lines represent cutoffs to determine IgA2+ and IgA1+ cells. B) Plot showing the average expression (nMFI) of IgA2 and the average number of IgA2+ cells in each cluster. The grey box highlights the three clusters showing high expression (nMFI) and the highest percentage of IgA2+ cells. C) UMAP coloured by the average pseudotime score calculate by Slingshot. D) Longitudinal expression (nMFI) of IgA2 and IgA1 in the activated switched memory (aSwM) B cells, early plasmablasts (PB) and plasmablasts from the CALIBRATE trial. Mean plus 95% confidence intervals are shown. Linear mixed model used to compare difference at 48 weeks between the two arms adjusting for baseline values. RB = belimumab after rituximab, R = rituximab. N = 38. nMFI= normalised mean fluorescence intensity. Only P values < 0.1 are shown.

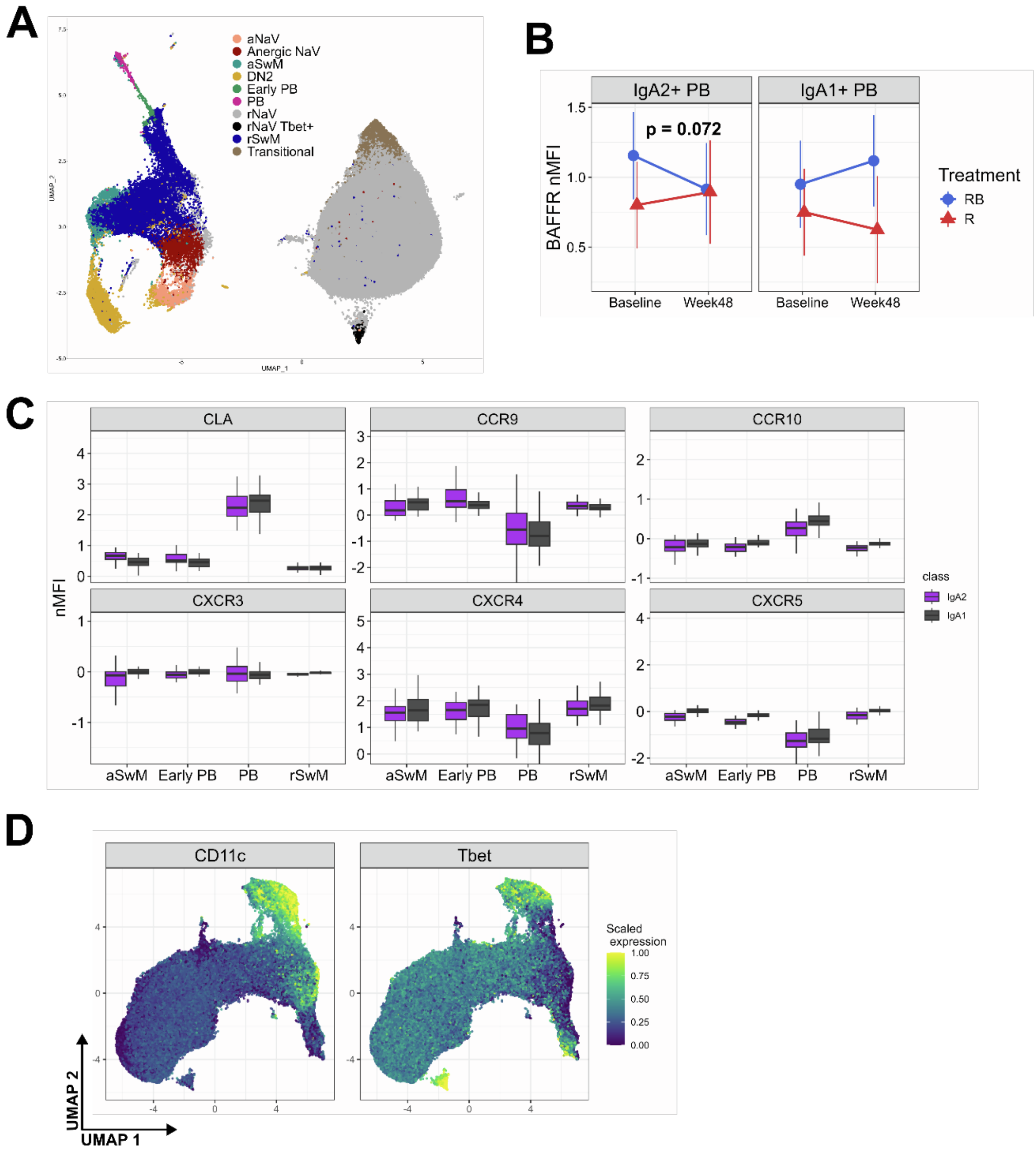

Supplementary Figure 4. BAFF and chemokine receptor expression in B cell subsets, related to Figures 3, 4 and 5

A) UMAP of clustered CD3-CD19+ B cells from 10 patients (from a cross-sectional lupus cohort, Table 2) with canonical subsets labelled as in Figure 1A. B) Longitudinal expression of BAFFR in IgA2+ and IgA1+ plasmablasts, showing the underlying data of Figure 3E. Mean plus 95% confidence intervals are shown. Linear mixed model used. C) Expression of chemokine receptors in IgA2+ and IgA1+ plasmablasts of CALIBRATE samples at baseline. Mean plus 95% confidence intervals are shown. D) UMAP of CD11c and Tbet expression in the CALIBRATE cohort. N = 10 for panel A. N = 38 for panel B-D. nMFI= normalised mean fluorescence intensity. Only P values < 0.1 are shown.

A

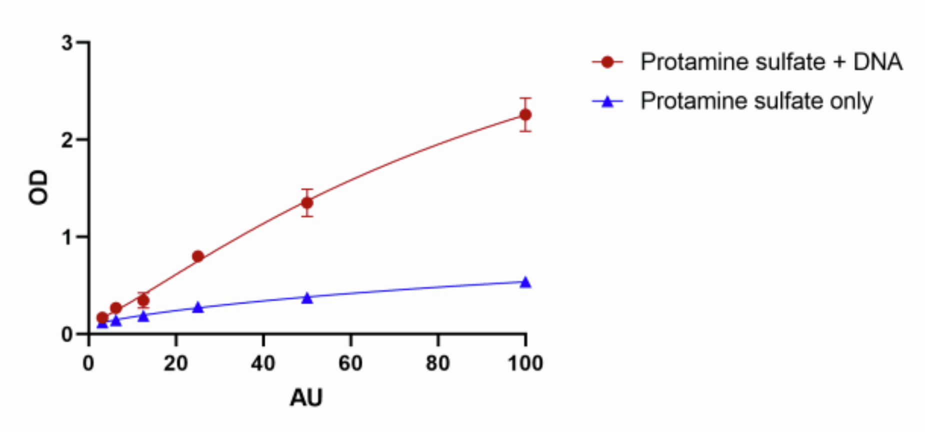

B

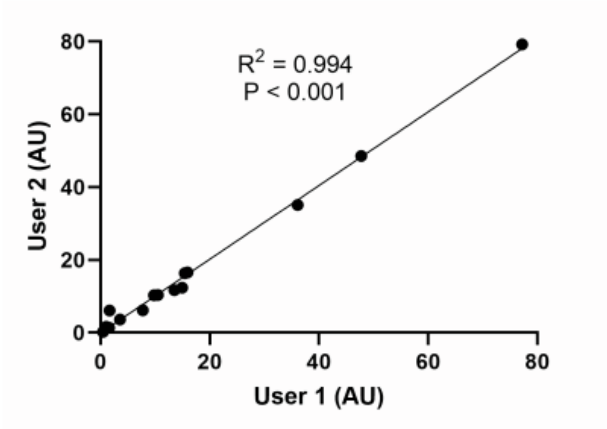

Supplementary Figure 5. Reproducibility and reliability of the IgA2 anti-dsDNA antibody ELISA, related to STAR Methods

A) Representative standard curve of the IgA2 anti-dsDNA positive control used for all samples. Values are the mean of two technical replicates. Red line shows binding to protamine sulfate and DNA, whilst blue line shows binding to protamine sulfate alone. Mean with 95% confidence intervals is shown. B) The arbitrary units (AU) obtained by two different users on the same serum samples (n=15) in two different, independent experiments. Pearson correlation with R^2 is shown.

Supplementary Table 1 | Baseline demographics and disease characteristics of the patients in the CALIBRATE trial, related to Figures 1-5

	RB (n = 19)	R (n =19)
Age, years ‡	34.5 (9.5)	32.4 (11.0)
Gender – no. of patients (%)		
Female	18 (95%)	15 (79%)
Ethnicity – no. of patients (%) §		
White	8 (42%)	6 (32%)
Black	8 (42%)	9 (47%)
Asian	2 (11%)	1 (5%)
Others or unknown as not reported	1 (5%)	3 (16%)
Concomitant immunosuppressant or prednisolone at screening, no. of patients (%)		
Mycophenolate	18 (95%)	16 (84%)
Azathioprine	6 (32%)	2 (10.5%)
Prednisolone	10 (53%)	13 (68%)
Receiving concomitant immunosuppressant or prednisolone	19 (100%)	19 (100%)
Concomitant hydroxychloroquine at screening, no. of patients (%)	14 (74%)	17 (89%)
Average daily prednisolone dose at screening, mg/day		
mean (SD)	30 (16.5)	37.7 (16.4)
median (IQR)	40 (21.2-40.0)	13 (20-40)
Patients taking ≥ 7.5 mg/day prednisolone, no. of patients (%) at screening	8 (42%)	13 (68%)
Patients taking ≥ 10 mg/day prednisolone at screening, no. of patients (%) at screening	8 (42%)	13 (68%)
Organ involvement (either BILAG-2004 A/B system scores) at screening, no. of patients (%)		
Constitutional	0 (0%)	0 (0%)
Cardiorespiratory	1 (5%)	1 (5%)
Mucocutaneous	4 (21%)	1 (5%)
Musculoskeletal	2 (11%)	2 (11%)
Neuro-psychiatric	1 (5%)	1 (5%)
Ophthalmic	0 (0%)	0 (0%)
Gastro-intestinal	1 (5%)	0 (0%)
Renal	19 (100%)	19 (100%)
Haematological	1 (5%)	0 (0%)
Renal Biopsy		
Class III/IV	8 (42%)	9 (47%)

Class V with class III/IV	11 (58%)	10 (53%)
Positive IgG anti-dsDNA antibody at screening, no. of patients (%)	18 (95%)	18 (95%)
IgG anti-dsDNA antibody level at screening, IU/ml		
mean (SD)	215 (110)	233 (106)
median (IQR)	301 (135-301)	301 (188-301)
Low complement C3 at screening, no. of patients (%)	9 (47%)	10 (53%)
Erythrocyte sedimentation rate (ESR) at screening,		
mean (SD)	3.6 (0.7)	3.6 (0.6)
median (IQR)	3 (3-4)	4 (3-4)
Lymphocyte count at screening, 10^9/L		
mean (SD)	1.3 (0.7)	1.3 (0.8)
median (IQR)	1 (1-2)	1 (1-1.8)
Platelet count at screening, 10^9/L		
mean (SD)	256 (90)	270 (88)
median (IQR)	236 (180-313)	283 (244-293)
Creatinine at screening (only renal BILAG-2004 A or B), micromol/L		
mean (SD)	107 (47)	107 (37)
median (IQR)	88.4 (88.4-88.4)	88.4 (88.4-88.4)
eGFR at screening (only renal BILAG-2004 A or B), ml/min/1.73 m^2		
mean (SD)	87.4 (35.1)	91.3 (38.6)
median (IQR)	90 (61.5 - 110.5)	86.5 (63 - 113.5)
Urine protein/creatinine ratio at screening, grams in 24 hours		
mean (SD)	3.4 (2.7)	3.4(1.8)
median (IQR)	3 (1-5)	3 (2-5)
Serum albumin at randomisation, g/L		
mean (SD)	30.5 (8.5)	28.9 (5.7)
median (IQR)	30 (30-40)	30 (30-40)

‡ *Mean (SD)*
§ *Reported by the patient*
†† *Screening refers to the first screening visit before rituximab*
IgG = Immunoglobulin G, IQR = Interquartile range, SD = standard deviation

Supplementary Table 2 | Baseline demographics and disease characteristics of the patients from the lupus cross-sectional cohort, related to Figures 3, 5 and 6.

	(n = 17)
Age, years ‡	34.2 (6.8)

Gender – no. of patients (%)	
Female	17 (100%)
Ethnicity – no. of patients (%) §	
White	9 (53%)
Black	3 (18%)
Asian	3 (18%)
Others or unknown as not reported	2 (12%)
Number of patients received rituximab within last 12 months, no. of patients (%)	7 (41%)
Concomitant immunosuppressant or prednisolone, no. of patients (%)	
Mycophenolate	9 (53%)
Azathioprine	1 (6%)
Prednisolone	6 (35%)
Receiving concomitant immunosuppressant or prednisolone	17 (100%)
Concomitant hydroxychloroquine, no. of patients (%)	15 (88%)
Average daily prednisolone dose, mg/day	
mean (SD)	6.5 (6.3)
median (IQR)	4.2 (2.1-5.2)
Patients taking ≥ 7.5 mg/day prednisolone, no. of patients (%)	8 (47%)
Patients taking ≥ 10 mg/day prednisolone at screening, no. of patients (%)	6 (35%)
Organ involvement (either BILAG-2004 A/B system scores), no. of patients (%)	
Constitutional	0 (0%)
Cardiorespiratory	1 (6%)
Mucocutaneous	7 (41%)
Musculoskeletal	13 (76%)
Neuro-psychiatric	0 (0%)
Ophthalmic	0 (0%)
Gastro-intestinal	0 (0%)
Renal	14 (82%)
Haematological	1 (6%)
Positive IgG anti-dsDNA antibody, no. of patients (%)	11 (65%)
IgG anti-dsDNA antibody level, IU/ml	
mean (SD)	125 (89)
median (IQR)	57 (35-104)

Low complement C3 at screening, no. of patients (%)	13 (76%)
Erythrocyte sedimentation rate (ESR) at screening,	
mean (SD)	13.8 (7.6)
median (IQR)	8.3 (3.3-16.5)
Lymphocyte count, 10^9/L	
mean (SD)	0.8 (0.8)
median (IQR)	0.7 (0.4-0.9)
Platelet count, 10^9/L	
mean (SD)	224 (109)
median (IQR)	212 (179-327)
Creatinine (only renal BILAG-2004 A or B), micromol/L	
mean (SD)	81.5 (28.2)
median (IQR)	78.1(71.1-82.5)
eGFR (only renal BILAG-2004 A or B), ml/min/1.73 m^2	
mean (SD)	89.7 (32.3)
median (IQR)	90(82.3 - 103.1)
Urine protein/creatinine ratio, mg/mmol	
mean (SD)	104 (112)
median (IQR)	89 (41-178)
Serum albumin at randomisation, g/L	
mean (SD)	34.7 (7.7)
median (IQR)	32.2 (30-42)
